# Beyond Disease: Happiness, Goals, and Meanings among Persons with Multiple Sclerosis and Their Caregivers

**DOI:** 10.3389/fpsyg.2017.02216

**Published:** 2017-12-20

**Authors:** Antonella Delle Fave, Marta Bassi, Beatrice Allegri, Sabina Cilia, Monica Falautano, Benedetta Goretti, Monica Grobberio, Eleonora Minacapelli, Marianna Pattini, Erika Pietrolongo, Manuela Valsecchi, Maria Pia Amato, Alessandra Lugaresi, Francesco Patti

**Affiliations:** ^1^Department of Pathophysiology and Transplantation, Università degli Studi di Milano, Milan, Italy; ^2^Department of Biomedical and Clinical Sciences “L.Sacco”, Università degli Studi di Milano, Milan, Italy; ^3^Neurology Unit, Multiple Sclerosis Centre, Ospedale di Vaio-Fidenza, Fidenza, Italy; ^4^Ospedale Policlinico Universitario G. Rodolico, Catania, Italy; ^5^Neurological Department, San Raffaele Hospital (IRCCS), Milan, Italy; ^6^Department of NEUROFARBA, University of Florence, Florence, Italy; ^7^Laboratory of Clinical Neuropsychology, ASST Lariana, Como, Italy; ^8^Department of Neurosciences, Imaging and Clinical Science, Università degli Studi ‘G. d'Annunzio’ Chieti - Pescara, Chieti, Italy; ^9^Department of Biomedical and Neuromotor Sciences, Università di Bologna, Bologna, Italy

**Keywords:** multiple sclerosis (MS), well-being, caregiving, daily living, meaning-making, goals, mixed method, psychosocial interventions

## Abstract

The experience of persons with multiple sclerosis (MS) and their caregivers is usually investigated in terms of emotional distress and health-related quality of life, while well-being indicators remain largely underexplored. In addition, findings are often interpreted from the clinical perspective, neglecting socio-cultural aspects that may crucially contribute to individuals' functioning. At the methodological level, most studies rely on scaled instruments, not allowing participants to freely express their needs and resources. Based on the bio-psycho-social perspective endorsed by the International Classification of Functioning, well-being indicators were investigated among 62 persons with MS (PwMS), their 62 caregivers and two control groups, matched by age and gender. Participants completed the Positive Affect Negative Affect Schedule (PANAS), the Satisfaction with Life Scale (SWLS), and the Eudaimonic and Hedonic Happiness Investigation instrument (EHHI). EHHI provides information on participants' happiness, goals and meanings through scaled and open-ended questions, contextualized within major life domains. No relevant differences emerged among PwMS and caregivers, compared with the respective control groups, as concerns life domains associated with happiness, goals and meaning. Participants across groups prominently mentioned family, highlighting its intrinsic value and its relevance as a sharing context; health did not represent a major theme for PwMS; community, society and religion/spirituality issues were substantially neglected by all participants. PwMS and caregivers reported lower levels of positive affect than their control groups, while no substantial differences emerged for negative affect, happiness and meaningfulness levels in life and across most domains. Results suggest that the experience of MS is associated with well-being in relevant life domains, such as family and close relationships. Although PwMS and caregivers identified a lower number of goals and meaning-related opportunities compared to control groups, they showed a positive adjustment to disease through the development of personal and family resources. These assets are often undervalued by health professionals and social institutions, while they could be fruitfully exploited through the active involvement of PwMS and their families as expert and exemplary informants in initiatives aimed at promoting the well-being of individuals and communities

## Introduction

The technological advancements in the medical domain fostered the representation and interpretation of health-related phenomena through rigorously quantitative approaches, leading to the affirmation of the biomedical model. Despite the shortcomings entailed by this narrow perspective, and the classical definition of health as “a state of complete physical, mental and social well-being” (World Health Organization, 1946, p. 1), both research and clinical practice are still permeated by a strictly biological view of human functioning.

A laudable effort to overcome this limitation and to promote the adoption of a bio-psycho-social view of health and disease is represented by the International Classification of Functioning, Disability and Health (ICF; World Health Organization, 2001). ICF is aimed at comprehensively assessing the physical, mental and social functioning of any individual, including persons with diseases and disabilities. It presents a marked shift in terminology, from focusing on the consequences of disease (impairments, disabilities, handicaps) to investigating the components of health and functioning, classified as structures and functions, daily activities, and social participation. Far from endorsing a pure linguistic convention, the ICF stems from a relevant conceptual change, leading to the evaluation of health conditions from a constructive perspective. It is based on the assumption of a dynamic interplay between individual features (body functions, activities, and participation) and environmental aspects that can facilitate or hinder the person's functioning (Dixon et al., 2008; Bodde and Seo, 2009). However, the implementation of this model into practice is problematic, as it requires a multidisciplinary integration effort involving researchers and practitioners from the healthcare, education and policy domains (Stucki et al., 2017). In addition, the assessment of psychosocial functioning through the current version of ICF poses several challenges. Psychological and environmental dimensions are collapsed into the heterogeneous domain of “contextual factors”; the psychological aspects are not explicitly identified through a checklist; and the components of community participation are difficult to evaluate (Chang et al., 2013). Despite these limitations, only the ICF currently offers an integrated approach to human functioning which can be used by professionals working in different sectors.

From the ICF perspective, it is not surprising that people with chronic diseases or disabilities describe themselves as ordinary persons who cope with extraordinary circumstances (Saravanan et al., 2001) that include biological impairments as well as material, social and institutional barriers. Despite these disadvantages, the mobilization of personal, relational and environmental resources allows these people to attain high levels of mental health (Arnold et al., 2005; Andrykowski et al., 2008; Delle Fave et al., 2015). Similarly, caregivers experience the coexistence of caring burden and limitations in daily opportunities with psychological and social resources (Song and Singer, 2006; Olsson et al., 2008; Fianco et al., 2015).

The study of mental health and well-being has received great impulse during the last two decades, through theories and empirical models deriving from two complementary perspectives. Within the hedonic perspective, well-being is operationalized as the predominance of positive over negative affect in daily experience and a globally positive life evaluation, defined as satisfaction with life (Ryan and Deci, 2001). From the eudaimonic perspective well-being is instead conceptualized as a dynamic growth process, that includes a wide range of constructs such as goal setting and pursuit, meaning-making, self-expressiveness, self-determination, self-acceptance, skill development and mastery, trust in relationships, and social integration (Huta and Waterman, 2014). Both these approaches proved to be useful in exploring protective resources, adaptation processes and adjustment outcomes that allow persons with chronic diseases and their caregivers to attain positive functioning (Cummins, 2005; Diener and Chan, 2011).

This avenue of research is however characterized by an emphasis on individual processes, while the societal and cultural factors influencing the person's daily functioning are substantially neglected (Di Martino et al., 2017). Demographic and contextual features, when investigated, are treated as components of the person's stable identity, despite the changes that both the environment and the individual ceaselessly undergo (Slife and Richardson, 2008).

### The individual and family experience of multiple sclerosis: challenges and resources

Multiple sclerosis (MS) is a chronic neurodegenerative disease, characterized by largely unpredictable symptoms and course, and currently lacking of curative treatment. Therefore, besides physical impairments, MS poses a number of psychological, behavioral and social challenges to both affected persons and their caregivers (Dennison et al., 2009; Ackroyd et al., 2011). The progressive course of the disease requires a constant re-adjustment over time (Bogosian et al., 2017), leading the person to gradually scale back or adapt daily activities to the new condition, find new occupations and interests, but also disengage from the adjustment process and withdraw from active life.

While the negative consequences of MS were extensively explored, research on the positive aspects is still limited. Studies have been conducted to investigate the eudaimonic process of meaning making, a key individual resource to attain well-being under unfavorable and irreversible conditions (Hicks and King, 2009). Meaning making allows individuals to integrate the problematic condition into a global and constructive view of their present and future life. The qualitative exploration of meaning making among persons with MS shed light on its components, which include disease acceptance, personal and relationship growth, and positive lifestyle changes (Pakenham, 2008a). Quantitative studies highlighted the positive association of sense making with life satisfaction, and its negative association with depression. Sense making was identified as a key predictor of positive adjustment also among caregivers (Pakenham, 2008b). Similar findings were detected as concerns perceived illness coherence, a construct closely related to meaning making; illness coherence was positively associated with eudaimonic and hedonic well-being indicators in both persons with MS and their caregivers (Bassi et al., 2016).

Benefit finding, consisting in the ability to identify positive consequences in an otherwise negative situation (Lechner et al., 2009), was also investigated among persons with MS and their family caregivers. Cross-sectional and longitudinal studies highlighted its positive association with meaning-based coping strategies, and its direct effects on positive adjustment outcomes, such as good dyadic relationships and subjective well-being (Pakenham, 2005, 2007, 2008b; Pakenham and Cox, 2009). Related findings support the idea of a “communal search for meaning” where persons with MS and their caregivers experience the trauma of a chronic illness and subsequently find positive aspects together (Pakenham, 2005). Further evidence of this dyadic process emerged from the investigation of post-traumatic growth and illness perceptions among persons with MS and their partners (Ackroyd et al., 2011).

These studies also showed that the disruption of life goals caused by MS progression can be counterbalanced by the development of new meanings and life purposes (Joseph and Linley, 2006). This process entails a complex interplay between disengagement from previous goals and reengagement in new ones (Neter et al., 2009). It also requires the implementation of adaptive tasks (Bensing et al., 2002), such as defining new challenges, acknowledging one's limits, maintaining emotional balance and self-esteem, facing uncertain future, cultivating social relationships, and looking at the bright side of life. Besides these individual resources, other factors located at the intersection between persons and their context may foster positive adaptation; they include facilitators of mobility/independence, social support, and social comparisons (Dilorenzo et al., 2008).

A frequent problem in studies investigating well-being and perceived quality of life among persons with MS is the lack of objective measures of disease severity; this limitation prevents researchers to exclude that positive adjustment may simply reflect a condition of less severe illness (Dennison et al., 2009). The assessment of demographic and clinical dimensions however entails methodological and interpretive challenges, as showed by the contrasting findings obtained in different studies. For example, in some studies demographic variables such as education and employment emerged as the strongest predictors of health-related quality of life (HR-QoL), together with clinical ones like depression and disability (Patti et al., 2003, 2007b). In other studies, HR-QoL itself emerged as an independent predictor of disability progression (Benito-Léon et al., 2013). Satisfaction with life was primarily predicted by disease severity and social support in one study (Ryan et al., 2007), while in another study age and education level emerged as the strongest predictors of hedonic and eudaimonic well-being (Bassi et al., 2014). Finally, a comparative study did not highlight significant differences in personal growth (a eudaimonic dimension) between persons with MS and healthy participants, while life satisfaction (a hedonic dimension) was significantly lower among the former (Barak and Achiron, 2011).

The investigation of well-being among caregivers of persons with MS is even less frequent, as studies are primarily focused on the emotional impact of caring-related burden and stress (Patti et al., 2007a; Rivera-Navarro et al., 2009), and on the daily choices and free time restrictions imposed by the caring role (Becker, 2011). Studies investigating satisfaction with life did not detect differences between caregivers of persons with MS and samples derived from the general population or carers of persons with other chronic diseases (Waldron-Perrine et al., 2009; Bassi et al., 2014). Eudaimonic well-being dimensions such as meaning making, benefit finding and illness coherence were more often investigated among caregivers, as reported in the previous paragraphs (Pakenham, 2007; Pakenham and Cox, 2009; Bassi et al., 2016). In a recent qualitative study involving Turkish caregivers (Topcu et al., 2016), participants mentioned both individual and social resources, such as motivation to care and perceived support.

Acknowledging the existence and adaptive role of well-being dimensions in the experience of MS surely represents an advancement. At the same time, most studies suffer from two conceptual limitations. The first one is the lack of a comprehensive bio-psycho-social perspective, and especially the neglect of the societal context surrounding persons with MS and their caregivers. Only few studies were conducted within the ICF framework. One of them was aimed at identifying a core set of ICF categories specific to MS that could be used in healthcare practice (Khan and Pallant, 2007). Another study proposed the Multiple Sclerosis Impact Profile (MSIP; Wynia et al., 2008) an instrument designed to evaluate the perceived impact of MS on functioning. A recent review (Dorstyn et al., 2017) highlighted the association between depressive symptoms and reduced social participation in persons with MS. A qualitative study exploring the impact of ICF contextual factors on the daily functioning of Jordanian persons with MS (Hamed et al., 2012) identified adequacy of financial and medical resources, religion and community awareness as facilitators, and social stigma as a barrier. Finally, the environmental and personal facilitators of social participation and satisfaction with parenting were investigated among mothers with MS (Farber et al., 2015). Findings suggested the importance of incorporating both categories of facilitators into treatment. The relevance of environmental factors also emerged from the investigation of unmet needs among persons with MS. These needs most often refer to contextual dimensions, such as family and social support, healthcare services, everyday life management, and the relationship with physicians (Galushko et al., 2014). Interestingly, representations of the unmet needs of persons with MS differ among health professionals (Golla et al., 2012): while physicians emphasize limitations in access to care due to poor financial resources, nurses and social workers are more aligned with patients' perceptions, quoting family support, social relationships and daily life management.

A second limitation characterizing the literature on MS is the emphasis on disease and related adjustment processes: participants and caregivers are rarely solicited to freely reflect on aspects of their life and daily experience potentially unrelated to disease or caregiving respectively. This limitation emerged in one of the studies investigating unmet needs of persons with MS (Galushko et al., 2014): participants expressed the need to be viewed as distinct individuals, not constrained by and identified through their health conditions.

### The present study

The first aim of the present study was to investigate different dimensions of well-being among persons with MS and their caregivers through a mixed method approach and from a bio-psycho-social, ICF informed perspective. Answers obtained through open-ended and scaled questions were jointly analyzed, in the attempt to contextualize findings within participants' global outlook of their own life and environmental opportunities. The well-being dimensions investigated in the study included happiness (its definition and recent related situations), hedonic well-being (positive and negative affect, satisfaction with life) and eudaimonic well-being (perceived meaningful things and goals). In addition, information was collected on happiness and meaningfulness levels in life in general, as well as in the specific domains of work, family, standard of living, interpersonal relations, health, personal growth, leisure, spirituality/religion, community, and society. Overall, the mixed method potentials are still underexploited in the psychological literature; moreover, to the best of our knowledge no studies have yet been conducted on these topics and through this methodological approach in the MS domain.

The second aim was to compare the findings collected among persons with MS and their caregivers with those obtained from two groups of participants, matched by age and gender, but with no history of chronic disease or caregiving experience. From an ICF informed perspective, this comparison was expected to shed light on group differences in perceived personal and contextual facets of well-being, including daily activities and opportunities, relational networks, and social participation. Attention was also paid to the role of employment status and education level in predicting well-being dimensions. These two demographic features represent crucial indicators of participation, classified in the ICF as contextual personal factors influencing human functioning (World Health Organization, 2002; Martins, 2015). The level of disability was also taken into consideration in the analysis of data collected among persons with MS.

Based on the available literature, some hypotheses were formulated. In line with studies on adjustment to MS (Pakenham, 2008a; Bassi et al., 2016) we expected that, when describing happiness, goals and meaningful things, a significantly higher percentage of persons with MS would refer to health compared with a control group. We also expected persons with MS to associate health with significantly lower levels of happiness and higher levels of meaningfulness, compared with a control group. As concerns caregivers, based on previous evidence (Pakenham, 2008b; Becker, 2011; Mausbach et al., 2011) we expected that a significantly higher percentage of participants would refer to family and a lower percentage to leisure, compared with a control group. We also expected caregivers to report significantly lower levels of happiness with leisure, and higher levels of meaningfulness in relation to family. As concerns ratings of affect and life satisfaction (the two components of hedonic well-being), the contrasting findings detected across studies (Barak and Achiron, 2011; Diener et al., 2017) did not allow us to formulate specific hypotheses. Finally, based on the literature highlighting the relevant role of education and employment in predicting individual well-being (Keyes, 2007; Patti et al., 2007b; Diener et al., 2017), we hypothesized that these two demographic features would provide a specific contribution to hedonic and eudaimonic well-being values across all groups. The same hypothesis was formulated as concerns the role of severity disease among persons with MS.

## Materials and method

### Participants

The study involved 248 Italian adults divided into four groups, each of them comprising 62 participants: persons with MS (PwMS), their caregivers, and two control groups of adults with no history of chronic disease or caregiving experience, selected from a larger study aimed at investigating well-being in the general population. Inclusion criteria for PwMS were being at least 18 years of age, having a clinically definite MS diagnosis for at least 3 years and having a caregiver; exclusion criteria comprised the presence of additional neurological or psychiatric disorders, severe cognitive impairment, MS in the active phase, and a condition of very severe disability, corresponding to a score above 8 on the Extended Disability Status Scale (EDSS; Kurtzke, 1983). The majority of PwMS involved in the study (59.68%) showed an EDSS score between 3.5 and 8 (indicating increasing levels of motor impairment), while 40.32% scored below 3.5 (indicating a high level of autonomy). No significant differences were detected between PwMS with low and high levels of disability for any of the variables examined in this study; therefore disability level will not be further considered in the following sections.

Caregivers were predominantly partners (59.67%) or parents (22.57%) of PwMS, siblings or children (each accounting for 6.46%), friends (3.23%), or professional caregivers (1 participant, 1.61%). Participants in the control groups were randomly extracted from a sample of 691 healthy Italian adults; age, gender and education level were used as filters to match the control groups with the respective comparison groups – PwMS and caregivers. It was however not possible to obtain a complete match between caregivers and their control group as concerns education level. The decision to include two different control groups was based on the significant age difference between PwMS and their caregivers (*t* = 2.85, *p* < 0.005). The demographic features of the four groups of participants are reported in Table 1.

**Table 1:** Demographic characteristics of the four groups.

	**PwMS**	**Control 1**	**Caregivers**	**Control 2**
	**(*N* = 62)**	**(*N* = 62)**	**(*N* = 62)**	**(*N* = 62)**
Age^a^	40.1 ± 9.7	40.5 ± 10.9	45.7 ± 12.0	45.7 ± 11.4
Age range	21–63	20–60	19–81	22–81
Gender (% Female)	69.35	69.35	58.06	56.45
**EDUCATION (%)**^b^				
High school or less	82.26	75.81	85.48	69.35
University	17.74	24.19	14.52	30.65
Work/study (%)	57.38	93.55	75.81	93.55
**CIVIL STATUS (%)**				
Married/cohabiting	66.13	51.61	77.42	72.13
Single/divorced/widowed	33.87	48.39	22.58	27.87

a *Means and Standard Deviations are reported*.

b *Education was dichotomized: “High school or less” includes elementary, middle, and high school; “University” includes graduation, post-graduation and PhD*.

No differences emerged between PwMS and their control group (control 1) for age, gender, marital status and education, while a significantly higher percentage of PwMS was unemployed (χ^2^ = 21.81, *p* < 0.001). As concerns caregivers and their control group (control 2), differences were detected for employment and education, both higher in the control group (χ^2^ = 7.52, *p* < 0.01, and χ^2^ = 4.61, *p* < 0.05 respectively).

### Materials

Data were collected through the following instruments:

A *socio-demographic questionnaire* provided information on participants' age, employment status, education level, and civil status. Clinical information including time from disease onset, level of disability, type of treatment and co-morbidities was collected for PwMS. Caregivers also reported their type of bond with PwMS.

*Eudaimonic and Hedonic Happiness Investigation* inventory (EHHI; Delle Fave et al., 2011, 2016). This mixed-method instrument allows researchers to collect qualitative and quantitative information on different components of well-being. Through open-ended questions, participants are asked to define happiness in their own words; to list the three future goals they consider most important, and the three things they consider most meaningful in their present life; and to briefly describe three situations associated with intense happiness during the last 6 months. In addition, participants are asked to rate on two sets of scaled items, ranging from 1 (extremely low) to 7 (extremely high), the levels of happiness and meaningfulness associated to 10 major life domains (work, family, standard of living, interpersonal relations, health, personal growth, leisure, spirituality/religion, community, and society) and to life in general.

*Satisfaction with Life Scale* (SWLS; Diener et al., 1985; Goldwurm et al., 2004). This widely used measure of well-being comprises five items on scales ranging from 1 (strongly disagree) to 7 (strongly agree). The items investigate the individuals' overall cognitive evaluation of their global life conditions and achievements.

*Positive Affect and Negative Affect Schedule* (PANAS; Watson et al., 1988; Terracciano et al., 2003). The instrument assesses the overall perceived intensity of positive and negative affect during daily life, through 10 items measuring components of positive affect (PA) and 10 items measuring components of negative affect (NA). Scales range from 1 (very slightly or not at all) to 5 (extremely).

### Procedure

This study involved seven different academic and clinical institutions; therefore, the protocol was submitted to the ethical committees of each institution. After approval from all committees, participants with MS and their caregivers were recruited at six MS centers in different Italian regions, in the context of a broader project aimed at investigating clinical, psychological and relational aspects of MS (Bassi et al., 2014, 2016). PwMS were contacted by the centers' personnel during check-ups or by phone, and were invited to identify their primary caregiver. Data from participants in the two control groups were selected from a larger study that had been approved by the ethical committee of the first author's institution.

Upon their expression of interest in joining the study, participants received detailed information on the project from a researcher involved in the study. They signed an informed consent in compliance with Italian privacy rules, and were provided with the battery of questionnaires. They could inspect the questionnaires, pose general and specific questions to the researcher, and express their doubts and concerns. PwMS and their caregivers were free to decide whether to complete the questionnaires immediately at the MS center, or at a time and place convenient to them. They could hand in their responses, or send them via mail. Before data processing researchers removed the consent form from each battery of questionnaires, thus guaranteeing participants' anonymity throughout the phases of data coding, storing and analysis. Data were stored in password protected computers. Participation to both the MS related study and the general survey was voluntary, and participants were free to leave the study at any time. Persons with MS were explicitly assured that refusal to participate or withdrawal from participation in the study would in no way interfere with the long-term healthcare services they were receiving at the MS center.

#### Coding procedure

Answers to the open-ended questions of the EHHI required an accurate coding work, using a coding system originally developed by Delle Fave et al. (2011), and gradually expanded through additions from various studies, in line with the bottom-up approach guiding the EHHI research project. In this coding system, answers are grouped into broad categories corresponding to the major life domains: work, family, standard of living, interpersonal relationships, health, personal/psychological life, leisure, spirituality/religion, society and community issues, and more general/unspecific life aspects. Multifaceted categories such as work, family, relationships, leisure, spirituality/religion, community/society and personal life are further subdivided into more fine-grained sub-categories (Delle Fave et al., 2013a). The categories family and interpersonal relationships were organized into the subcategories intrinsic value (e.g., having a family; a partner; children; friends), sharing (e.g., spending time with children; sharing life projects with partner; sharing good and bad experiences with friends), personal contribution (caring for elder parents; raising children; helping friends in need), family well-being/harmony, balance (e.g., health of family members; children's positive development; harmony in relationships) and personal reward (e.g., gratification from partner, children; gratitude expressions from siblings; satisfaction with relations). A similar partition characterizes community/society issues. In the psychological/personal life category, some subcategories refer to eudaimonic well-being dimensions (growth/engagement, purpose, competence/mastery, autonomy, self-actualization, meaning/value, harmony/balance, fullness/awareness, optimism), and others to hedonic well-being ones (satisfaction/achievement, positive emotions, and absence of negative feelings); a less specific subcategory, labeled as “positive experiences/states”, includes answers generically referring to happiness as “inner well-being”, “a stable state”, or “a way of being.”

The coding procedure for each answer comprises first the identification of a category in which the answer can be included, and then a specific numeric item to which the answer can correspond, if available. If an answer content does not fit any available item, a new item will be added to the category. As concerns happiness definitions, participants often report complex descriptions including different components of happiness. Each of these facets is treated as a specific semantic unit and coded separately; up to six answer units are retained for each participant. Based on the specific formulation of the questions concerning goals, meaningful things, and recent situations of most intense happiness, three answer units are retained for each question. In the present study, reliability in the coding process was established through the involvement of two expert coders providing independent ratings for each answer; divergences were clarified through a subsequent discussion; unsolved discrepancies and answers potentially requiring the inclusion of new items in the coding system were further discussed with the first author.

As concerns the scaled questions included in the EHHI, and the data collected through PANAS and SWLS, the numeric values corresponding to the perceived levels of each variable were reported. The EHHI items assessing the levels of happiness and meaningfulness associated with personal/psychological life are labeled as “personal growth”; this decision was originally based on the assumption that growth and development could best represent the positive and meaningful sides of this life domain (Delle Fave et al., 2013a).

#### Statistical analyses

The analysis of qualitative data collected through the EHHI was aimed at identifying the life domains predominantly mentioned by participants across groups in their descriptions of happiness, major goals, meaningful things, and recent sources of intense happiness. Results were compared between groups (respectively PwMS and control 1, and caregivers and control 2). Since a high percentage of participants across groups indicated family as prominent goal, meaningful thing, and source of recent happiness, analyses were performed on family sub-categories. The same in-depth analysis was conducted for the psychological definitions of happiness.

Coded answers were first grouped into the corresponding categories and sub-categories; subsequently, considering that each participant provided more than one answer for each question, the number of participants reporting at least one answer in each category and subcategory was calculated across groups. This approach allowed us to compare the percentage of participants referring to each answer category and subcategory between groups through 2 × 2 frequency tables by means of the χ^2^ procedure. The procedure was not considered as reliable when the number of participants in one or more cells was below 5. Through Spearman coefficients, correlations were then calculated between participants' distribution in answer categories and their demographic and group features. Logistic regression analyses allowed us to verify whether demographic or group features predicted a specific pattern of answers.

Quantitative data were first analyzed using descriptive statistics. Correlations of hedonic and eudaimonic dimensions of well-being with participants' group and demographic features were calculated through Pearson and Spearman coefficients. Hierarchical regression analyses provided information on the specific contribution of participants' group and demographic features to well-being dimensions.

Given the large number of group comparisons performed on quantitative and qualitative variables, we took a Bonferroni approach, adjusting the critical alpha value for significance to the number of *t*-tests and χ^2^ comparisons performed on the same dataset (25 and 61 respectively). More specifically, to achieve α < 0.05 with 25 *t*-tests, the alphas obtained from each data set had to score below 0.002 (α < 0.05/25, two-tailed); in order to reject a null hypothesis, the test statistic had to exceed critical *t* = 3.16, corresponding to *p* < 0.002 with 122 degrees of freedom (for two groups, df = *N* participants-2).

The same approach was used for frequency table comparisons (*N* = 61 in each data set). To achieve α < 0.05 with 61 comparisons, the alphas obtained from the data set had to score below 0.0008 (α < 0.05/61); in order to reject a null hypothesis, the test statistic had to exceed critical χ^2^ = 11.34, corresponding to *p* < 0.0008 with 1 degree of freedom, since df = (N columns-1) (N rows-1). As for correlations, regardless of the statistical significance only coefficient values equal to or higher than 0.30 were considered as adequate to interpret associations as meaningful (Hinkle et al., 2003). More specifically, values between 0.30 and 0.50 indicated low correlation, values between 0.50 and 0.70 moderate correlation, and values above 0.70 high correlation.

## Results

This section illustrates the qualitative and quantitative findings presented for each group separately, in order to allow for comparisons.

### Qualitative findings

The findings obtained from the open-ended questions of the EHHI are presented as percentages of participants across groups who provided at least one answer in the different categories and subcategories. The answers referring to psychological dimensions are grouped into the category labeled as “psychological definitions” for the definitions of happiness, and into the category “personal life” for goals, meaningful things and recent situations of intense happiness.

#### Happiness definitions

Participants were invited to answer the question “What is happiness for you?” in their own words, without specific constraints. It is worth noticing that 9 PwMS and 8 caregivers either did not provide any answer, or stated that “happiness does not exist,” while only 2 participants in each control group did so. Moreover, control groups 1 and 2 reported on average a higher number of answer units in their happiness definitions (2.65 and 2.80 respectively) compared with PwMS and caregivers (2.25 and 2.19 respectively). These differences were however not statistically significant. Table 2 shows for each group the percentage distribution of the participants who provided at least one answer in the different categories and subcategories. Results of group comparisons are also reported.

**Table 2:** Definitions of happiness: percentage of participants mentioning each category and subcategory by group, and comparisons between groups.

	**PwMS**	**Control 1**	**χ^2^**	**Caregivers**	**Control 2**	**χ^2^**
Psychological definitions	54.72	93.33	22.51^**^	66.67	80.00	2.61
Harmony/balance	68.97	60.71	0.56	63.89	72.92	0.78
Other eudaimonic	24.14	39.29	1.95	25.00	41.67	2.52
Hedonic	41.38	46.43	0.19	27.78	33.33	0.30
Positive states	10.34	10.71		2.78	8.33	
Family	45.28	15.00	12.48^*^	51.85	28.33	6.58
Intrinsic value	28.00	11.11		13.79	11.76	
Sharing	48.00	55.56	0.69	44.83	58.82	0.83
Personal contribution	4.00	11.11		10.34	0	
Family well-being	12.00	22.22		34.48	23.53	
Personal reward	28.00	22.22		20.69	23.53	
Interpersonal relations	15.09	23.33	1.22	12.96	30.00	4.82
Health	11.32	8.33	0.29	12.96	15.00	0.10
Work	11.32	1.67		5.56	8.33	
Standard of living	1.89	1.67		7.41	3.33	
Leisure	5.66	0		0	1.67	
Spirituality, Religion	3.77	1.67		3.70	6.67	
Community, Society	7.55	5.00		0.00	3.33	
Life in general	0	13.33		3.70	8.33	
N participants^a^	53	60		54	60	

a Each participant could provide more than one answer; Bonferroni adjusted alpha

* p < 0.05;

** *p < 0.01; χ^2^-values are not reported if the no. of participants in one or more cells falls below 5*.

Overall, a substantial similarity emerged between groups across most categories. Only two significant differences were detected between PwMS and control 1: a lower percentage of PwMS reported psychological definitions of happiness, and a lower percentage of controls reported family-related ones. Despite these overarching differences, the percentage distribution of participants across subcategories of both psychological and family-related definitions did not differ between the two groups. Among psychological definitions, participants predominantly referred to eudaimonic constructs, such as harmony/balance, self-actualization, personal growth, and optimism. Since a remarkably high percentage of participants specifically mentioned harmony/balance, related findings are reported separately in Table 2. As concerns family, most participants in both groups provided answers related to sharing (experiences, activities, projects). Personal rewards followed as subcategory; answers included receiving love expressions, support, acknowledgment from family members, and satisfaction with family. Fewer participants quoted the other domains, and a negligible percentage in both groups mentioned leisure, spirituality/religion, community/society issues and standard of living. A similar answer pattern was detected among caregivers and control 2, but no significant group differences emerged.

#### Goals and meaningful things

While all participants in the control groups identified some important future goals, 2 PwMS and 4 caregivers did not. In addition, 59 participants in control 1 reported up to three goals compared with 45 PwMS (χ^2^ = 11.68, *p* < 0.05); similarly, 59 participants in control 2 reported up to three goals compared with 44 caregivers (χ^2^ = 12.89, *p* < 0.05). Table 3 depicts the percentage distribution of the participants who provided at least one answer across categories in each group.

**Table 3:** The most important future goals: percentage of participants mentioning each answer category by group and comparisons between groups.

	**PwMS**	**Control 1**	**χ^2^**	**Caregivers**	**Control 2**	**χ^2^**
Personal life	26.67	38.71	2.00	15.52	20.97	0.59
Family	68.33	67.74	0.004	79.31	64.52	3.23
Intrinsic value	51.22	52.38	0.01	39.13	40.00	0.007
Sharing	14.63	4.76		10.87	25.00	2.96
Personal contribution	12.20	23.81	1.89	23.91	12.50	1.84
Family well-being	31.71	35.71	0.15	43.48	45.00	0.02
Personal reward	4.88	0		0	2.50	
Interpersonal relations	5.00	6.45		1.72	6.45	
Health	53.33	22.58	12.28^*^	39.66	32.26	0.71
Work	45.00	59.68	2.63	37.93	58.06	4.86
Standard of living	6.67	16.13	2.69	29.31	22.58	0.71
Leisure	10.00	19.35	2.12	8.62	16.13	1.54
Spirituality, Religion	3.33	0.00		3.45	6.45	
Community, Society	3.33	3.23		0.00	14.52	
Life in general	3.33	12.90		6.90	14.52	
N participants^a^	60	62		58	62	

a Each participant could provide more than one answer; Bonferroni adjusted alpha

* *p < 0.05; χ^2^-values are not reported if the no. of participants in one or more cells falls below 5*.

The majority of participants in all groups mentioned family, specifically referring to its intrinsic value (having a family; having or adopting children; having grandchildren; finding the right partner) and to the well-being of family members (physical health, self-actualization or goal achievement of children, grandchildren, partner, siblings, and parents). Health, work, and personal life were mentioned by lower percentages of participants; spirituality/religion and community/society were marginally represented, together with interpersonal relations. The only significant difference concerned the higher percentage of PwMS mentioning health, compared to control 1.

As regards the most meaningful things in the present life, 7 caregivers did not identify any, compared to 4 PwMS and no participants in the control groups (χ^2^ = 13.22, *p* < 0.05). Moreover, 60 participants in control 2 reported up to three meaningful things, compared with only 46 caregivers (χ^2^ = 12.74, *p* < 0.05). Nevertheless, as illustrated in Table 4, participants' percentage distribution across answer categories was largely overlapping across groups.

**Table 4:** The most meaningful things in present life: percentage of participants mentioning each answer category by group and comparisons between groups.

	**PwMS**	**Control 1**	**χ^2^**	**Caregivers**	**Control 2**	**χ^2^**
Personal life	12.07	20.97	1.71	25.45	17.74	1.03
Family	91.38	79.03	3.58	83.64	82.26	0.04
Intrinsic value	86.79	83.67	0.20	78.26	84.31	0.59
Sharing	1.89	12.24		13.04	5.84	
Personal contribution	1.89	6.12		6.52	3.92	
Family well-being	7.55	0		6.52	7.84	
Personal reward	7.55	2.04		2.17	1.96	
Interpersonal Relations	27.59	40.32	2.16	23.64	38.71	3.06
Health	48.28	30.65	3.90	32.73	33.87	0.02
Work	43.10	30.65	2.00	43.64	50.00	0.47
Standard of living	5.17	12.90		9.09	14.52	
Leisure	5.17	17.74		3.64	9.68	
Spirituality, Religion	8.62	6.45		5.45	9.68	
Community, Society	5.17	11.29		3.64	1.61	
Life in general	0	13.33		5.45	4.84	0
N participants^a^	58	62		55	62	

a *Each participant could provide more than one answer; χ^2^-values are not reported if the no. of participants in one or more cells falls below 5*.

Participants in all groups almost unanimously quoted family as one of the most meaningful things in their lives (specifically referring to its intrinsic value); progressively lower percentages of participants mentioned work, health, interpersonal relationships and personal life, without significant group differences. Spirituality/religion, leisure, standard of living and community/society were mentioned by less than 10% of the participants across groups.

#### Recent situations of intense happiness

As illustrated in Table 5, only 39 caregivers (62.9%) provided answers to this question, while the remaining 23 (37.10%) could not remember any recent situation of intense happiness. This answer distribution pattern was significantly different from those detected for the other three groups (χ^2^ = 29.93, *p* < 0.001): only 12 participants (19.35%) among PwMs, 5 (8.1%) in control 2 and 2 (3.23%) in control 1 did not report recent situations of intense happiness.

**Table 5:** Recent situations of intense happiness: percentage of participants mentioning each answer category by group and comparisons between groups.

	**PwMS**	**Control 1**	**χ^2^**	**Caregivers**	**Control 2**	**χ^2^**
Personal life	6.00	8.33		0	10.53	
Family	74.00	73.33	0.006	82.05	78.95	0.14
Intrinsic value	10.81	22.73	1.99	12.50	15.56	
Sharing	70.27	68.18	0.04	56.25	46.67	0.69
Personal contribution	0	4.55		6.25	0	
Family well-being	35.14	29.55	0.29	50.00	55.56	0.23
Personal reward	13.31	13.64		3.13	11.11	
Interpersonal relations	22.00	36.67	2.79	20.51	22.81	0.07
Health	18.00	3.33	6.52	5.13	1.75	
Work	20.00	21.67	0.05	12.82	29.82	3.79
Standard of living	4.00	6.67		7.69	10.53	
Leisure	34.00	31.67	0.08	38.46	33.33	0.27
N participants^a^	50	60		39	57	

a *Each participant could provide more than one answer; χ^2^-values were not reported if one or more cells included less than 5 participants; answer categories cited by less than 5 participants across groups were excluded from analysis*.

No group differences instead emerged in the percentage of participants who provided at least one answer across categories. Family ranked first again; within this category, the majority of participants across groups referred to sharing positive events and experiences, such as anniversaries and other celebrations. A lower percentage of participants mentioned events promoting the well-being of family members (positive school and work achievements, disease recovery or health improvements). Leisure predominantly included the practice of arts and crafts, sports, travels and media fruition. Interpersonal relationships and work followed in rank across groups. A higher percentage of PwMS referred to health related situations, but after Bonferroni adjustment the difference with control 1 was not significant. Spirituality/religion and community/society were again reported by a negligible percentage of participants across groups, together with personal life and standard of living.

### Quantitative findings

#### Ratings of happiness, meaningfulness, and hedonic well-being

Table 6 shows the mean ratings of happiness and meaningfulness across groups, and the results of the comparisons between PwMS and control 1, and between caregivers and control 2.

**Table 6:** Levels of happiness and meaningfulness in life domains and comparisons between groups.

	**PwMS *M* (*sd*)**	**Control 1 *M* (*sd*)**	***t***	**Caregivers *M* (*sd*)**	**Control 2 *M* (*sd*)**	***t***
**HAPPINESS**						
Work	4.50 (1.96)	4.47 (1.31)	0.11	4.25 (1.72)	4.56 (1.25)	1.13
Family	5.90 (1.47)	5.60 (1.34)	1.18	5.56 (1.35)	5.84 (1.20)	1.13
Standard liv.	4.74 (1.34)	4.90 (1.30)	0.69	4.41 (1.28)	4.88 (1.28)	2.00
Relations	5.00 (1.55)	5.10 (1.34)	0.37	4.70 (1.42)	5.33 (1.06)	2.75
Health	4.37 (1.72)	5.45 (1.10)	4.17^**^	5.21 (1.14)	5.54 (1.30)	1.40
Personal Growth	5.05 (1.41)	4.92 (1.35)	0.52	4.87 (1.43)	5.18 (1.16)	1.31
Leisure	4.15 (1.48)	4.66 (1.45)	1.96	3.98 (1.72)	4.41 (1.66)	1.41
Spirituality	3.93 (2.09)	4.04 (1.70)	0.29	4.02 (2.04)	4.89 (1.56)	2.60
Community	3.64 (1.41)	4.23 (1.44)	2.18	3.56 (1.67)	4.49 (1.31)	3.29^*^
Society	3.71 (1.60)	3.62 (1.43)	0.29	3.70 (1.73)	3.72 (1.20)	0.08
Life in general	5.03 (1.31)	5.08 (1.12)	0.22	5.10 (1.25)	5.31 (0.93)	1.05
**MEANINGFULNESS**						
Work	5.28 (1.76)	5.21 (1.29)	0.24	5.36 (1.67)	5.66 (1.23)	1.12
Family	6.45 (1.08)	6.58 (0.82)	0.75	6.72 (0.77)	6.69 (0.82)	0.19
Standard liv.	5.15 (1.34)	5.03 (1.19)	0.50	4.93 (1.28)	5.29 (1.19)	1.60
Relations	5.56 (1.35)	6.02 (0.98)	2.13	5.34 (1.28)	5.97 (0.96)	3.10
Health	6.32 (1.02)	6.44 (0.84)	0.67	6.53 (0.88)	6.81 (0.44)	2.19
Personal Growth	5.74 (1.25)	5.91 (1.08)	0.85	5.51 (1.37)	5.90 (1.31)	1.64
Leisure	4.87 (1.52)	5.10 (1.17)	0.93	4.55 (1.42)	5.55 (1.04)	4.48^**^
Spirituality	4.01 (2.12)	4.00 (2.12)	0.04	4.61 (2.06)	5.08 (1.79)	1.35
Community	4.26 (1.37)	4.45 (1.67)	0.70	4.19 (1.60)	4.64 (1.45)	1.63
Society	4.29 (1.54)	4.25 (1.62)	0.14	4.26 (1.68)	4.88 (0.99)	2.53
Life in general	5.98 (1.06)	6.08 (0.80)	0.57	5.90 (1.25)	6.27 (0.85)	1.93
N participants	62	62		62	62	

Bonferroni adjusted alpha

* p < 0.05;

** *p < 0.01*.

Participants across groups reported the highest levels of happiness and meaningfulness in the domains of family, health, interpersonal relations, life in general, and personal growth (with slight variations in domain order across groups). In contrast, spirituality/religion, community and society issues were associated with the lowest levels of happiness and meaningfulness across groups. The only difference between PwMs and control 1 concerned happiness with health, with the former reporting significantly lower values. The comparison between caregivers and control 2 highlighted two significant differences: Caregivers reported higher values of happiness in relation to community issues, and they attributed lower meaningfulness to the domain of leisure.

More relevant differences were detected between groups as concerns the hedonic well-being dimensions. As illustrated in Table 7, both PwMS and caregivers scored significantly lower on positive affect compared with their respective control groups. Satisfaction with life ratings were significantly lower among PwMS than among participants in control 1. No group differences were instead detected for negative affect.

**Table 7:** Levels of affective and cognitive dimensions of hedonic well-being, and their comparison between groups.

	**PwMS *M* (*sd*)**	**Control 1 *M* (*sd*)**	***t***	**Caregivers *M* (*sd*)**	**Control 2 *M* (*sd*)**	***t***
Positive affect	3.01 (0.81)	3.59 (0.57)	4.62^**^	3.01 (0.71)	3.87 (0.48)	7.83^**^
Negative affect	2.15 (0.93)	2.51 (0.82)	2.29	2.03 (0.89)	2.22 (0.66)	1.35
Satisfaction with life	3.80 (1.55)	4.65 (1.30)	3.30^*^	4.14 (1.51)	4.87 (1.15)	3.06
N participants	62	62		62	62	

Bonferroni adjusted alpha

* p < 0.05;

** *p < 0.01*.

#### Demographic and group predictors of well-being dimensions

Correlations and regression models were finally calculated within each group set-PwMS and control 1, and caregivers and control 2 respectively -, in order to investigate the role of group type and demographic features as predictors of qualitative and quantitative evaluations of well-being. Age, gender and marital status were not considered, as these characteristics did not differ between PwMS and control 1, and between caregivers and control 2. Attention was instead paid to education level and employment status, as group differences were detected for these two features, as reported in the comments on Table 1. Table 8 illustrates Spearman correlations among predictors (demographics and group classification) and between predictors and well-being dimensions.

**Table 8:** Correlations among demographic and group predictors, and between predictors and variables showing significant differences in the two group sets.

	**Employment**	**Education**	**Group**
**PwMS/CONTROL 1**			
Employment	–	0.11	0.42^***^
Education	0.11	–	0.08
Positive affect	0.28^**^	0.22^*^	0.39^***^
Negative affect	−0.004	−0.15	0.20^*^
Satisfaction with life	0.35^***^	0.24^**^	0.29^**^
Happiness with health	0.31^*^	0.05	0.33^*^
Happiness def.—psychological	0.16	0.13	0.49^***^
Happiness def.—family	−0.04	−0.09	−0.29^**^
Goals—health	−0.19^*^	−0.03	−0.30^***^
**CAREGIVERS/CONTROL 2**			
Employment	–	0.12	0.25
Education	0.12	–	0.19^*^
Positive affect	0.26^**^	0.30^***^	0.58^***^
Negative affect	0.11	0.03	0.12
Satisfaction with life	0.16	0.15	0.27^**^
Happiness with community	0.06	0.21^*^	0.30^**^
Leisure meaningfulness	0.23^**^	0.12	0.38^***^

* p < 0.05;

** p < 0.01;

*** *p < 0.001. “Group” is a dummy variable with 1 for PwMS or caregivers and 0 for the respective control groups*.

For the sake of synthesis, analyses were performed only for the dimensions showing significant differences between groups. As reported in the method section, correlations higher than 0.30 were deemed as meaningful. Overall, group classification showed the most numerous and strongest correlations with the well-being dimensions. In the PwMS/control 1 group set, only employment status showed a low positive correlation with satisfaction with life; together with group classification, it showed a low positive correlation with happiness with health as well. No meaningful correlations were instead identified for education level. In the caregivers/control 2 group set, group type showed low to moderate correlations with most well-being dimensions, except for satisfaction with life and negative affect. A low positive correlation emerged between education level and positive affect, while employment status did not show any relevant correlation with well-being dimensions.

Based on these results, linear regressions with stepwise selection were carried out to investigate the role of employment status, education level and group classification as predictors of the hedonic well-being dimensions: positive affect, negative affect, and satisfaction with life. In addition, linear regressions (with scaled outcome variables) and binary logistic regressions (with categorical outcome variables) were performed to assess the role of demographic features in the findings obtained through the EHHI for which group differences were identified. More specifically, for the PwMS/control 1 group set regressions were conducted for happiness with health, psychological and family-related definitions of happiness, and health-related goals. For the caregivers/control 2 group set regressions were conducted for happiness with community and meaningfulness of leisure.

As concerns PwMS and control 1, for the dependent variable positive affect the two predictors entered in the model were group type and education level, together explaining 18% of the variable variance (*r*^2^ = 0.143, *F* = 20.13, *p* < 0.001, and *r*^2^ = 0.039, *F* = 5.41, *p* < 0.05 respectively). Negative affect was significantly predicted by group type (*r*^2^ = 0.041, *F* = 5.27, *p* < 0.05), however explaining only 4.4% of the variable variance. Satisfaction with life was significantly predicted by employment status and education level (*r*^2^ = 0.121, *F* = 16.69, *p* < 0.001, and *r*^2^ = 0.042, *F* = 5.82, *p* < 0.05 respectively), but not by group type; the two significant predictors together explained 16.3% of the variable variance. The level of happiness with health was predicted by group type (*r*^2^ = 0.126, *F* = 17.47, *p* < 0.001), and employment status (*r*^2^ = 0.048, *F* = 7.04, *p* < 0.01), together explaining 17.4% of the variable variance. The distribution of participants citing psychological and family-related happiness definitions was predicted by group type only (*B* = −1.27, OR = 0.079, Wald χ^2^ = 22.63, *p* < 0.001; and *B* = 0.67, OR = 3.820, Wald χ^2^ = 9.04, *p* < 0.01 respectively). Group type was also the sole predictor of health-related goals distribution (*B* = 0.66, OR = 3.783, Wald χ^2^ = 11.20, *p* < 0.001).

In the caregivers/control 2 group set, positive affect was significantly predicted by group type and education level, together explaining 36.9% of the variable variance (*r*^2^ = 0.334, *F* = 61.25, *p* < 0.001, and *r*^2^ = 0.035, *F* = 6.75, *p* < 0.05 respectively); no significant predictors were instead identified for negative affect. Group type emerged as the only significant predictor for the three remaining variables: satisfaction with life (*r*^2^ = 0.071, *F* = 9.36, *p* < 0.01, explaining 2.2% of the variable variance); happiness with community (*r*^2^ = 0.088, *F* = 10.80, *p* < 0.01, explaining 2.6% of the variable variance) and leisure meaningfulness (*r*^2^ = 0.141, *F* = 20.04, *p* < 0.001, explaining 4.2% of the variable variance).

## Discussion

This study aimed at investigating different facets of well-being among persons with MS and their caregivers from an integrated perspective. At the conceptual level, the eudaimonic dimensions of goal pursuit and meaning-making were jointly investigated with the hedonic ones of affect and life satisfaction. In addition, based on a bio-psycho-social and ICF informed perspective, the investigation of well-being was contextualized within the major life domains, and findings were compared with those reported by two groups of participants sharing similar demographic features, but not exposed to experiences of chronic illness or caregiving. At the empirical level, the adoption of a mixed method approach gave participants the opportunity to freely describe their present evaluations and future expectations.

### The private context of well-being: a shared perspective

The information collected through the EHHI allowed us to contextualize individuals' perceived well-being within the major life domains. Regardless of group inclusion, in both qualitative and quantitative answers participants primarily referred to few life domains, basically circumscribed to the private sphere. Family, personal life and health distinctly emerged across groups as the predominant contexts of happiness, meaningfulness and goal investment. A substantially lower relevance was attributed to the broader contexts of work and interpersonal relations; finally, the public domains of community and society, and the transcendent sphere of spirituality/religion were almost absent from participants' qualitative answers, and they scored lowest in rank on the scales. Overall, these findings largely confirmed previous EHHI studies conducted across countries on adult samples belonging to the general population (Delle Fave et al., 2011, 2013b).

Across groups, participants who referred to family in their definition of happiness mainly focused on the dimension of sharing. This finding is consistent with the models emphasizing the primacy of relational connectedness in humans (Richardson, 2012). As specifically concerns persons with MS and their caregivers, these results are also consistent with the evidence of a shared process of adaptation to illness, based on communal growth and search for meaning (Pakenham, 2005; Pakenham and Cox, 2009; Ackroyd et al., 2011). Among the psychological definitions of happiness, eudaimonic dimensions were predominant; inner harmony was reported by the highest percentage of participants across groups, followed by self-actualization and personal growth. This finding, consistent with previous evidence (Delle Fave et al., 2016), provides further support to the view of happiness as connectedness, at both the inner and relational levels (Kjell, 2011). Most of the research hypotheses concerning happiness definitions were however not confirmed. Against expectations, the percentage of PwMS referring to health and the percentage of caregivers referring to family were not significantly higher, compared to their respective control groups. Similarly, against expectations no difference was detected between caregivers and control 2 in the percentage of participants quoting leisure. Only two unexpected differences emerged between PwMS and control 1: a lower percentage of PwMS provided psychological definitions of happiness, and a higher percentage mentioned family related ones. In both cases, group classification emerged as the only significant predictor. The overall relevance of family has been widely documented among persons with MS (Ryan et al., 2007; Pakenham, 2008a). It is however worth noting that in this study persons with MS, similarly to participants in the other groups, emphasized sharing rather than receiving support and rewards from family—as it could be expected from individuals facing a progressively increasing dependence on their caregivers.

Across groups, the majority of participants mentioned family as a major future goal, primarily referring to its intrinsic value and to the well-being of family members. Work followed in rank, while marginal relevance was attributed to extra-family relations, and to social, community and spirituality issues. As hypothesized, a significantly higher percentage of persons with MS quoted health, compared to control 1; group classification was the only predictor of this result. All the other hypotheses were not confirmed. It is worth noticing that, compared to their respective control groups, a significantly higher percentage of PwMS and caregivers reported less than three future goals. This result can be related to the perceived uncertainty highlighted in the MS literature, and in studies involving people with other chronic diseases (Bensing et al., 2002; Tams et al., 2016). Uncertainty leads individuals to focus on the present rather than on future planning; this aspect can be even more relevant in diseases such as MS, which entails a higher margin of unpredictability, compared with other chronic and degenerative conditions (Alschuler and Beier, 2015).

Family also emerged as a key meaningful thing; the vast majority of participants across groups referred to its intrinsic value—having a family, a partner, children, siblings were identified as valuable components of life *per se*. The primacy of intimate relationships in the meaning making process largely confirmed previous evidence obtained with a variety of samples (Baumeister and Leary, 1995; Lambert et al., 2010; Taubman – Ben-Ari et al., 2012). Work, health and interpersonal relations followed across groups, while only a negligible percentage of participants mentioned spirituality/religion, community/society, and leisure. A significantly higher percentage of caregivers reported less than three meaningful things compared to control participants; this difference may be related to the narrower range of daily opportunities that characterizes the caring role (Mausbach et al., 2011).

When describing recent situations of intense happiness, most participants across groups referred to events shared with the family: birthdays, marriages, holidays, but also receiving a good news concerning family members. Leisure and interpersonal relationships, following in rank, were mentioned by a relatively high percentage of participants across groups only in the context of this answer. Finally, and in line with expectations, health was quoted almost exclusively by PwMS. It is also important to note that over one third of the caregivers could not identify any recent situation of intense happiness.

### Extraordinary circumstances, ordinary experiences of well-being

Research has repeatedly emphasized the negative psychological consequences of living with MS as a person or a caregiver, especially at the emotional level. Our findings were only partially consistent with the literature, rather emphasizing the “ordinariness” of PwMS and caregivers (Saravanan et al., 2001) in their quantitative ratings of global and domain-related well-being.

As concerns domain-related happiness and hedonic well-being (assessed as positive and negative affect, and satisfaction with life), PwMS scored significantly lower than control participants in happiness with health (thus confirming expectations) and in positive affect. Although participants' group emerged as the strongest predictor of the two variables, unemployment further contributed in predicting lower happiness with health, and lower education level in predicting lower positive affect. PwMS also reported significantly lower levels of life satisfaction, but employment status and education, rather than presence of MS, emerged as significant predictors of this result. No differences instead emerged between PwMS and control participants for negative affect, despite the (weakly) significant predictive role of group classification. To this purpose, it is worth mentioning that negative affect values were on average higher in the control group. The comparison between caregivers and their control group highlighted a significant difference in positive affect, with the former scoring significantly lower. Group type was the strongest predictor of positive affect; the additional though limited contribution of lower education level replicated the findings detected between PwMS and control 1. Group type emerged as significant predictor of satisfaction with life as well, though with marginal explanatory relevance.

The lower levels of positive affect reported by PwMS and caregivers were consistent with the literature highlighting the emotional burden of chronic disease; nevertheless, education emerged in both cases as an additional environmental predictor, suggesting that emotions are—at least partially—socially constructed. In particular, this finding provides support to the role of education as a major objective indicator of hedonic or subjective well-being, regardless of health conditions (Kroll and Delhey, 2013). The same consideration can be made as regards satisfaction with life, whose levels were not predicted by group type, but by the social opportunities derived from employment status and education, thus confirming previous evidence (Oishi and Diener, 2014). The lack of group differences in negative affect levels confirmed instead the conceptual and empirical independence of positive and negative affect (Seib-Pfeifer et al., 2017), as well as the importance to consider well-being and ill-being as partially independent domains of experience, rather than as opposite poles of a single continuum (Keyes, 2007).

In this study eudaimonic well-being was quantitatively assessed as the level of meaningfulness associated with different life domains. Findings did not support the study hypotheses, as PwMS and caregivers did not differ from control groups in family and health related meaningfulness. An unexpected difference emerged between caregivers and control 2, with the former attributing significantly lower meaningfulness to the leisure domain. This result, solely predicted by participants' group, can be related to the restrictions in daily life opportunities experienced by family caregivers (Becker, 2011). Since under these circumstances leisure activities get often sacrificed first, downplaying their meaning and relevance can help caregivers adjust to the related constraints (Pakenham and Cox, 2009).

### Patients and caregivers: social assets beyond clinical labels

This study highlighted that overall persons with MS and their caregivers do not differ from healthy people in their experience of hedonic and eudaimonic well-being. These findings are consistent with the literature showing that individuals and families mobilize a variety of resources in order to adjust to disease conditions. At the same time, they offer further suggestions. Not only have these people adjusted to disease; they pursue value-driven goals, cultivate inner harmony and balance, invest their energies in meaningful activities and relationships. Persons with MS do not seem to be primarily focused on their own health and related needs; they are rather actively engaged in sharing experiences and collaborating to the promotion of family well-being (Bogosian et al., 2017). As for caregivers, their life trajectory—although forcefully disengaged from extra-family socialization and leisure—is grounded in personal and relational values, despite the costs emerging at the hedonic well-being level. In general, the present study did not highlight group differences in participants' level of engagement in public roles and social activities, thus showing that active involvement in community and society is related to cultural dimensions rather than health conditions.

Overall, these findings can be considered as a provocative claim for a change in perspective, as concerns the social representation of health. This claim, consistent with the ICF model, is based on theoretical and empirical evidence. As highlighted by studies investigating resilience (Walsh, 2015), individuals and families experiencing chronic diseases should be valued and appreciated for their ability to develop personal and communal competences, rather than being considered as weak and low-performing members of the society. Their psychological and relational competences, laboriously built over time, could be rather shared to the benefit of others. Community Based Rehabilitation (CBR) programs are rooted in this view, aimed at empowering persons with disabilities through the promotion and acknowledgment of their active community role (Khasnabis et al., 2010). Across countries, people experiencing disease are often founders or active members of associations, promoters of fundraising campaigns in support of biomedical research, civil rights activists. Therefore, health professionals could approach them as experts who can offer first-person knowledge of a specific condition, and not only as patients to treat and caregivers to instruct (Greenhalgh, 2009). Their social involvement could be extended to educational programs and other community initiatives, allowing them to share their resources and enjoy recognition as full-fledged members of the society. Although this change implies an overall revision of the health culture, the advantages would be remarkable, as efforts in this direction could lead to a more inclusive and participative society.

### Study strengths and limitations

The major strength of the present study is the investigation of hedonic and eudaimonic dimensions of well-being among persons with MS, their caregivers and two control groups through a mixed method approach. The complexity of this research design, too often neglected by researchers (Morales-Gonzales et al., 2004), allows for contextualizing scaled ratings within qualitative, semantically richer answers. In this study, the domain-related ratings of happiness and meaningfulness could be combined with a fine grained description of the same domains, their present and future relevance, and their relation to well-being. The findings provided an integrated representation of the daily activities, contexts and relationships in which participants' meaning-making process, goal pursuit and happiness experiences took place. To our knowledge, no studies of this kind are available in the psychological literature on MS, and more generally on chronic diseases.

This study has limitations as well. First of all, the cross-sectional design prevented from identifying causal relationships among variables. Although the circumscribed observation field allowed for an in-depth analysis of well-being, the negative impact of disease and caregiving was not explored. The sample sizes were relatively small. Disability levels of the persons with MS did not reflect the whole range of progression stages: the inclusion of participants with very severe disability could lead to different results, at both the qualitative and quantitative levels. All participants were Italians, thus belonging to a specific socio-cultural context: this feature increases reliability in the comparison of results across samples, but it prevents from generalizing results to countries characterized by different healthcare, welfare and value systems.

### Future directions

The findings from this study shed light on participants' experience of well-being, in the context of their daily activities and social roles. It is however important to consider that both individuals and their contexts are dynamic entities, changing over time while interacting with each other; this further level of complexity, endorsed by the ICF model, can be satisfactorily evaluated only through the collaboration of researchers from different disciplinary fields. As specifically concerns psychology, relevant contributions could derive from community and cultural psychology, with their focus on the interaction dynamics between socio-cultural practices, individual experience and collective behaviors (Christopher and Hickinbottom, 2008; Di Martino et al., 2017).

A stronger interdisciplinary collaboration is especially needed in the light of a specific result emerged from this study: persons with MS, their caregivers and the control groups reported low levels of happiness and meaningfulness in community and society issues; in addition, these domains were almost absent from their lists of goals, meaningful things, and occasions for happiness. This finding may be interpreted as an alarming sign of civic disengagement. However, it confirms evidence obtained in other studies conducted in individualistic societies (Delle Fave et al., 2011). We consider this result as a general warning for researchers, practitioners and policy makers, highlighting the pressing need to promote a culture of interconnectedness (Prilleltensky, 2005), in order to contrast the deterioration of community networks presently emerging across nations.

## Author contributions

ADF and MB provided substantial contributions to the conception and design of the work; the acquisition, analysis, and interpretation of data; drafting the work and revising it critically for important intellectual content; final approval of the version to be published; and agreement to be accountable for all aspects of the work in ensuring that questions related to the accuracy or integrity of any part of the work are appropriately investigated and resolved. All the other co-authors provided substantial contributions to the acquisition and interpretation of data; critical revision of the work at the conceptual level; final approval of the version to be submitted; and agreement to be accountable for all aspects of the work in ensuring that questions related to the accuracy or integrity of any part of the work are appropriately investigated and resolved.

### Conflict of interest statement

There was no involvement of study sponsors in the study design; collection, analysis and interpretation of data; writing of the manuscript; decision to submit the manuscript for publication. ADF, MB, BA, MP, SC, MF, EM, MG, MV, MPA, and EP declare that the research was conducted in the absence of any commercial or financial relationships that could be construed as a potential conflict of interest. BG received personal grant for speaking and advisor activities from Biogen and TEVA. FP received personal grant for speaking and advisor activities from Almirall, Bayer, Biogen, Celgene, Merck, Novartis, Roche, Sanofi, TEVA. MPA received research grants and honoraria as a speaker and member of advisory boards from Bayer, Biogen, Merck, Novartis, Roche, Sanofi Genzyme and Teva. AL served as Advisory Board Member for Bayer, Biogen, Genzyme/Sanofi, Merck Serono, Novartis, Teva; this author also received travel grants and honoraria from Bayer, Biogen, Genzyme, Merck Serono, Novartis, Sanofi, and Teva, as well as travel and research grants from Associazione Italiana Sclerosi Multipla; this author's institution received research grants from Almirall, Bayer, Biogen, Merck Serono, Novartis, Sanofi, and Teva.
